# A study of clinical and serological correlation of early myocardial injury in elderly patients infected with the Omicron variant

**DOI:** 10.3389/fcvm.2024.1268499

**Published:** 2024-02-14

**Authors:** Xueying Yu, Xiaoguang Li, Shuai Xia, Lu Lu, Jiahui Fan, Ying Wang, Yan Fu, Chen Suo, Qiuhong Man, Lize Xiong

**Affiliations:** ^1^ Department of Clinical Laboratory, Shanghai Fourth People’s Hospital, School of Medicine, Tongji University, Shanghai, China; ^2^ Department of Thyroid, Breast and Vascular Surgery, Shanghai Fourth People’s Hospital, School of Medicine, Tongji University, Shanghai, China; ^3^ Key Laboratory of Medical Molecular Virology (MOE/NHC/CAMS), Shanghai Institute of Infectious Disease and Biosecurity, School of Basic Medical Sciences, Shanghai Frontiers Science Center of Pathogenic Microbes and Infection, Fudan University, Shanghai, China; ^4^ Department of Epidemiology and Ministry of Education Key Laboratory of Public Health Safety, School of Public Health, Fudan University, Shanghai, China; ^5^ Shanghai Key Laboratory of Anesthesiology and Brain Functional Modulation, Translational Research Institute of Brain and Brain-Like Intelligence, Shanghai Fourth People’s Hospital, School of Medicine, Tongji University, Shanghai, China; ^6^ Department of Anesthesiology and Perioperative Medicine, Shanghai Fourth People’s Hospital, School of Medicine, Tongji University, Shanghai, China

**Keywords:** Omicron variant, elderly, myocardial injury, clinical characteristics, risk factors

## Abstract

**Introduction:**

Myocardial injury in elderly Omicron variant patients is a leading cause of severe disease and death. This study focuses on elucidating the clinical characteristics and potential risk factors associated with myocardial injury in elderly patients infected with the Omicron variant.

**Methods:**

Myocardial injury was defined based on elevated cardiac troponin concentrations exceeding the 99th percentile upper reference limit. Among 772 elderly Omicron-infected patients, categorized into myocardial injury (*n* = 263) and non-myocardial injury (*n* = 509) groups. The stratified log-rank statistic was used to compare the probability of patients developing intensive care. Receiver operating characteristic curves were used to determine the best cut-off values of clinical and laboratory data for predicting myocardial injury. Univariate and multivariate logistic regression was adopted to analyze the risk factors for myocardial injury.

**Results:**

The occurrence of myocardial injury in Omicron variant-infected geriatric patients was up to 34.07% and these patients may have a higher rate of requiring intensive care (*P* < 0.05). By comparing myocardial injury patients with non-myocardial injury patients, notable differences were observed in age, pre-existing medical conditions (e.g., hypertension, coronary heart disease, cerebrovascular disease, arrhythmia, chronic kidney disease, and heart failure), and various laboratory biomarkers, including cycle threshold-ORF1ab gene (Ct-ORF1ab), cycle threshold-N gene (Ct-N), white blood cell count, neutrophil (NEUT) count, NEUT%, lymphocyte (LYM) count, LYM%, and D-dimer, interleukin-6, procalcitonin, C-reactive protein, serum amyloid A, total protein, lactate dehydrogenase, aspartate aminotransferase, glomerular filtration rate, blood urea nitrogen, and serum creatinine (sCr) levels (*P *< 0.05). Furthermore, in the multivariable logistic regression, we identified potential risk factors for myocardial injury in Omicron variant–infected elderly patients, including advanced age, pre-existing coronary artery disease, interleukin-6 > 22.69 pg/ml, procalcitonin > 0.0435 ng/ml, D-dimer > 0.615 mg/L, and sCr > 81.30 μmol/L.

**Conclusion:**

This study revealed the clinical characteristics and potential risk factors associated with myocardial injury that enable early diagnosis of myocardial injury in Omicron variant-infected elderly patients, providing important reference indicators for early diagnosis and timely clinical intervention.

## 1 Introduction

The severe acute respiratory syndrome coronavirus 2 (SARS-CoV-2) poses a major threat to the respiratory system. It has been shown that SARS-CoV-2 can also invade other organs such as the heart, kidneys, liver, and blood vessels. In particular, heart damage is a common complication in patients with novel coronavirus SARS-CoV-2 infections. Endothelial cells, cardiomyocytes, and pericytes in the heart express angiotensin-converting enzyme angiotensin 2 (ACE2) receptors, transmembrane serine protease 2, and furinase, which form the structural basis for SARS-CoV-2 invasion (1, 2). Direct damage to cardiomyocytes from SARS-CoV-2 infection is possible. In addition, the cytokine storm and systemic inflammatory response triggered by SARS-CoV-2 can cause cardiac tissue injury. More serious cardiovascular complications such as arrhythmias, myocardial infarction, and heart failure may then occur (3).

Since December 2021, different SARS-CoV-2 Omicron variants have caused several global epidemics (4). The exceptional infectivity of the Omicron variant has resulted in rapid population spread and has led to a large number of infections. Previous clinical studies reported that myocardial injury was the most common complication in patients infected with the wild-type SARS-CoV-2, and the occurrence of myocardial injury has been associated with poor prognosis and outcomes, including admission to the intensive care unit and death (5–7). However, the clinical characteristics and risk factors for myocardial injury in elderly patients infected with the Omicron variant have yet to be fully understood. Therefore, in this study, we retrospectively analyzed clinical and laboratory biomarkers from elderly patients infected with the Omicron variant at the Fourth People's Hospital of Tongji University, Shanghai, with the aim of identifying the clinical characteristics and risk factors associated with early myocardial injury in these patients to facilitate clinical risk stratification and improve their clinical management.

## 2 Methods

### 2.1 Study design and participants

In April 2022, the Fourth People's Hospital of Shanghai, Tongji University, was designated as one of the hospitals responsible for treating patients infected with the Omicron variant during the outbreak of the epidemic in Shanghai. In this study, a cohort of 772 patients was included out of a total of 2,645 patients recorded in the medical system between 10 April and 27 June 2022. The inclusion criteria comprised confirmed cases of the SARS-CoV-2 Omicron variant, individuals aged 60 and above, those with TnI test results within 24 h of hospitalization, and those with complete medical information. Exclusion criteria applied to unconfirmed cases of the Omicron variant, individuals under 60 years of age, those without TnI results within 24 h of hospitalization, and those lacking medical information. The study was conducted following the 1975 Declaration of Helsinki (8), was approved by the Ethics Committee of the Fourth People's Hospital of Tongji University (No. 2022097-001), and was published in the Chinese Clinical Trials Registry (CHiCTR2200065440). Written informed consent was waived by the ethics committee of the designated hospital for patients during the SARS-CoV-2 Omicron variant pandemic.

### 2.2 Data collection

Two researchers retrieved demographic characteristics (age and gender), clinical data (symptoms, comorbidities, treatment methods, complications, and outcomes), and laboratory findings from each patient admitted to the hospital using the hospital's electronic medical records database. Data for all the clinical biomarkers in this study were collected immediately after the admission of the patients.

According to the Fourth Universal Definition of Myocardial Infarction guidelines, myocardial injury is defined as a serum level of the cardiac biomarker TnI above 99% of the upper reference limit (5, 6). In this study, the serum level of TnI was measured in patients admitted to the hospital within the first 24 h. The patients were classified according to the presence or absence of myocardial injury; a level above 0.028 ng/ml is defined as an abnormal serum TnI level according to test specifications.

The clinical course classification and treatment was in accordance with the ninth version of the national COVID-19 guidance (9). To confirm the presence of infection, 2019-nCoV detection kits (Shanghai Bojin Medical Technology Company) were used to detect the cycle threshold (Ct) of the ORF1ab gene (Ct-ORF1ab) and the N gene (Ct-N) of nasopharyngeal swab samples using real-time reverse transcriptase-polymerase chain reaction. Both the Ct-N gene and Ct-ORF1ab greater than 35 were judged as negative. Mild cases are defined as those with mild symptoms such as fever, fatigue, and loss of taste/smell but without radiographic evidence of pneumonia. Moderate cases refer to those with typical symptoms of respiratory infection (e.g., fever, dry cough, fatigue) and radiographic evidence of pneumonia. Severe cases refer to those patients with at least one of the following conditions: breathing problems, low oxygen saturation, low PaO_2_/FiO_2_ (PaO_2_ denotes a partial pressure of oxygen in arterial blood; FiO_2_ denotes the fraction of inspired oxygen), or progressive symptoms combined with pulmonary imaging showing an obvious progress of lesions (>50%) within 24–48 h. Patients who meet any of three criteria, namely, respiratory failure, shock, or organ failure requiring admission to an intensive care unit, are considered severe cases. In this study, we combined severe and critical patients into a severe group for statistical analysis.

Other laboratory indicators, including data on white blood cell (WBC, 10^9^/L) count, neutrophil (NEUT, 10^9^/L) count, neutrophil% (NEUT%), lymphocyte (LYM, 10^9^/L) count, lymphocyte% (LYM%), monocyte (MON, 10^9^/L) count, monocyte% (MON%), hemoglobin (g/L), C-reaction protein (CRP, mg/L), procalcitonin (ng/ml), serum amyloid A (SAA, mg/L), interleukin-6 (pg/ml), myoglobin (ng/L), creatinine kinase-myocardial band (CK-MB, ng/L), N-terminal pro-B-type natriuretic peptide (NT-proBNP, pg/ml), total protein (TP, g/L), lactate dehydrogenase (LDH, U/L), aspartate aminotransferase (AST, U/L), alanine aminotransferase (ALT, U/L), glomerular filtration rate (estimated) (eGFR), blood urea nitrogen (BUN), and serum creatinine (sCr), were collected from patients who were admitted to the hospital within the first 24 h.

### 2.3 Statistical methods and analyses

Descriptive statistics were obtained for all study variables. Statistical analysis results of continuous variables were described as the mean ± standard deviation (SD) if they had a Gaussian distribution, as determined using the Kolmogorov–Smirnov test. Otherwise, they were expressed as the median and interquartile range (IQR). Categorical variables were compared using Pearson's chi-squared test, but in the case of limited data, Fisher's exact test was used. Continuous variables were compared using *t*-tests or Mann–Whitney *U* tests, as appropriate. The stratified log-rank statistic was used to compare the probability of patients requiring intensive care. The optimal statistical cutoff values for distinguishing myocardial injury were calculated based on the receiver operating characteristic (ROC) curve. Considering the power analysis, there are 26 concomitant variables in our study; the number of myocardial injury cases required was 260 based on the 10 EPV (10 events per variable) method. Logistic regression was employed to analyze potential risk factors for the occurrence of myocardial injury. All statistical analyses were performed using SPSS 20 or R v4.1.3, and all graphs were drawn using GraphPad. The significance threshold for all analyses was set at *P* < 0.05.

## 3 Results

### 3.1 Clinical characteristics of Omicron variant–infected elderly patients with and without myocardial injury

**Figure 1:**
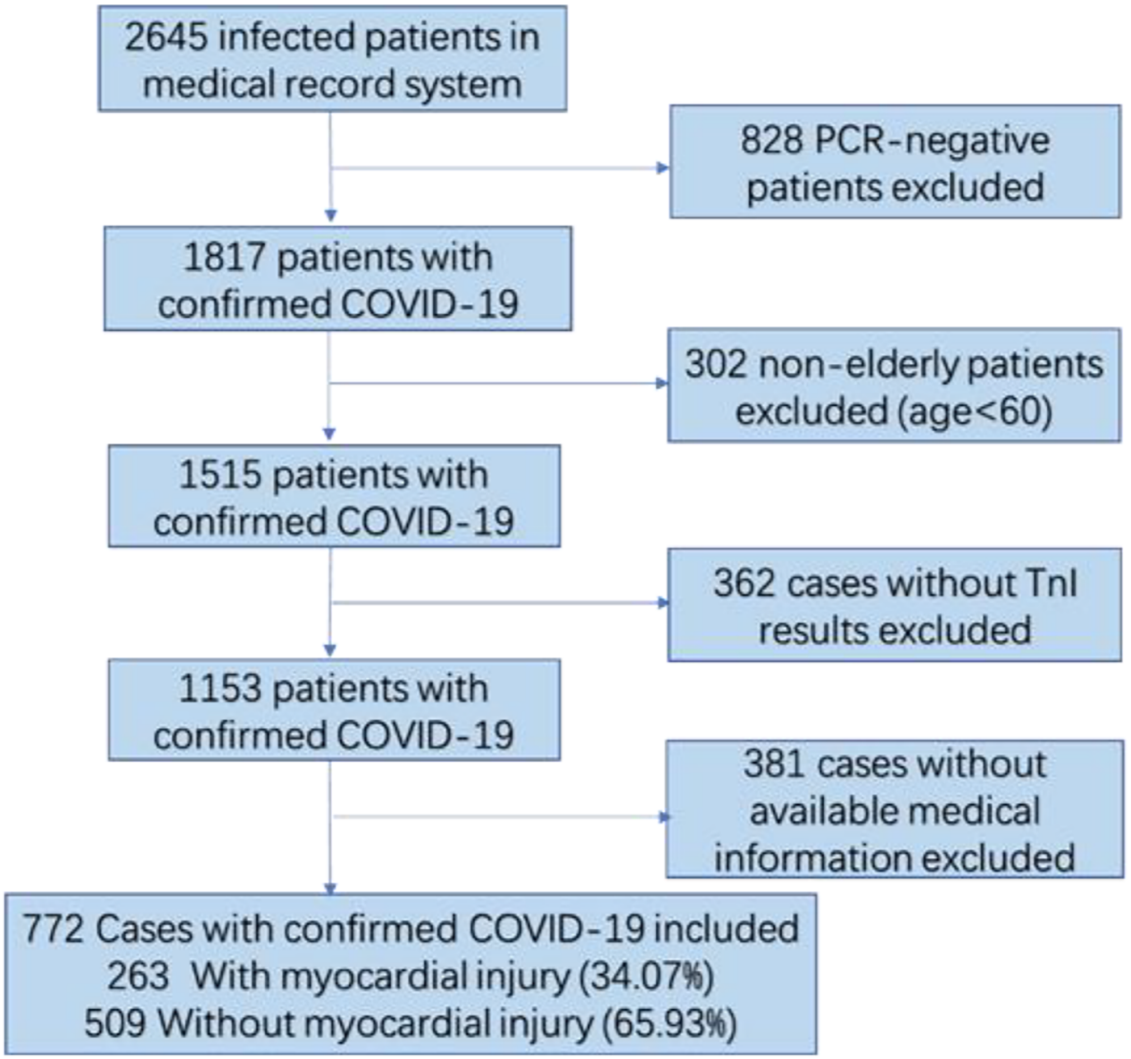
A flowchart of participant selection.

Out of a total of 2,645 patients recorded in the medical system between 10 April and 27 June 2022, we excluded 828 patients whose SARS-CoV-2 Omicron variant status was not confirmed, 302 patients aged below 60 years, 362 patients who lacked TnI test results within the initial 24 h of hospitalization, and 381 patients with unavailable medical information. Consequently, a cohort of 772 patients was included in this study. Figure 1 depicts the patient recruitment process.

In the enrolled population, the median age was 82 years (range, 60–104 years), with 326 (42.23%) patients being men. Among the cohort, 263 patients (34.07%) had a myocardial injury, while 509 patients (65.93%) did not have a myocardial injury (Table 1). Among these patients, the most common symptoms were cough [458 patients (59.33%)], sputum production [339 patients (43.91%)], and fever [199 patients (25.78%)]. Sore throat, shortness of breath, rhinorrhea, and chest tightness were present in 102 (13.21%), 66 (8.55%), 43 (5.57%), 37 (4.79%), and 19 (4.6%) patients, respectively. Dizziness [21 (2.72%) patients], headache [19 (2.46%) patients], and myalgia [16 (2.07%) patients] were rare. Hypertension [471(61.01%) patients], coronary artery disease [232 (30.05%) patients], cerebrovascular disease, including cerebral hemorrhage and cerebral infarction [218 (28.24%) patients], and diabetes [191 (24.74%) patients] were the most common pre-existing diseases. The proportions of arrhythmia, tumor, chronic kidney disease, chronic obstructive pulmonary disease, and heart failure were 12.31% (*n* = 95), 11.01% (*n* = 85), 7.12% (*n* = 55), 2.33% (*n* = 18), and 2.07% (*n* = 16), respectively.

**Table 1:** Demographics and clinical characteristics of elderly patients with and without myocardial injury who were infected with the Omicron variant.

	Patients, No. (%)			
		Myocardial injury		
Characteristic	Total (*n* = 772)	Present (*n* = 263)	Absent (*n* = 509)	*P*-value
Demographics				
Age, median (range), years	82 (60–104)	87 (61–104)	78 (60–101)	<0.001
Female	446 (57.77%)	155 (58.94%)	291 (57.17%)	0.638
Signs and symptoms at admission				
Cough	458 (59.33%)	154 (58.56%)	304 (59.72%)	0.754
Sputum production	339 (43.91%)	115 (43.73%)	224 (44.01%)	0.940
Fever	199 (25.78%)	68 (25.86%)	131 (25.74%)	0.971
Sore throat	102 (13.21%)	30 (11.41%)	72 (14.15%)	0.287
Shortness of breath	66 (8.55%)	33 (12.55%)	33 (6.48%)	0.004
Rhinorrhea	43 (5.57%)	13 (4.94%)	30 (5.89%)	0.585
Chest tightness	37 (4.79%)	19 (7.22%)	18 (3.54%)	0.023
Dizziness	21 (2.72%)	10 (3.80%)	11 (2.16%)	0.184
Headache	19 (2.46%)	6 (2.28%)	13 (2.55%)	0.817
Muscle ache	16 (2.07%)	2 (0.76%)	14 (2.75%)	0.066
Previous medical history				
Hypertension	471 (61.01%)	184 (69.96%)	287 (56.39%)	<0.001
Coronary artery disease	232 (30.05%)	117 (44.49%)	115 (22.59%)	<0.001
Cerebrovascular disease	218 (28.24%)	92 (34.98%)	126 (24.75%)	0.003
Diabetes	191 (24.74%)	57 (21.67%)	134 (26.33%)	0.156
Arrhythmia	95 (12.31%)	47 (17.87%)	48 (9.43%)	0.001
Neoplasm	85 (11.01%)	21 (7.98%)	64 (12.57%)	0.054
Chronic kidney disease	55 (7.12%)	34 (12.93%)	21 (4.13%)	<0.001
Chronic pulmonary disease	18 (2.33%)	6 (2.28%)	12 (2.36%)	0.947
Heart failure	16 (2.07%)	12 (4.56%)	4 (0.79%)	<0.001

**Figure 2:**
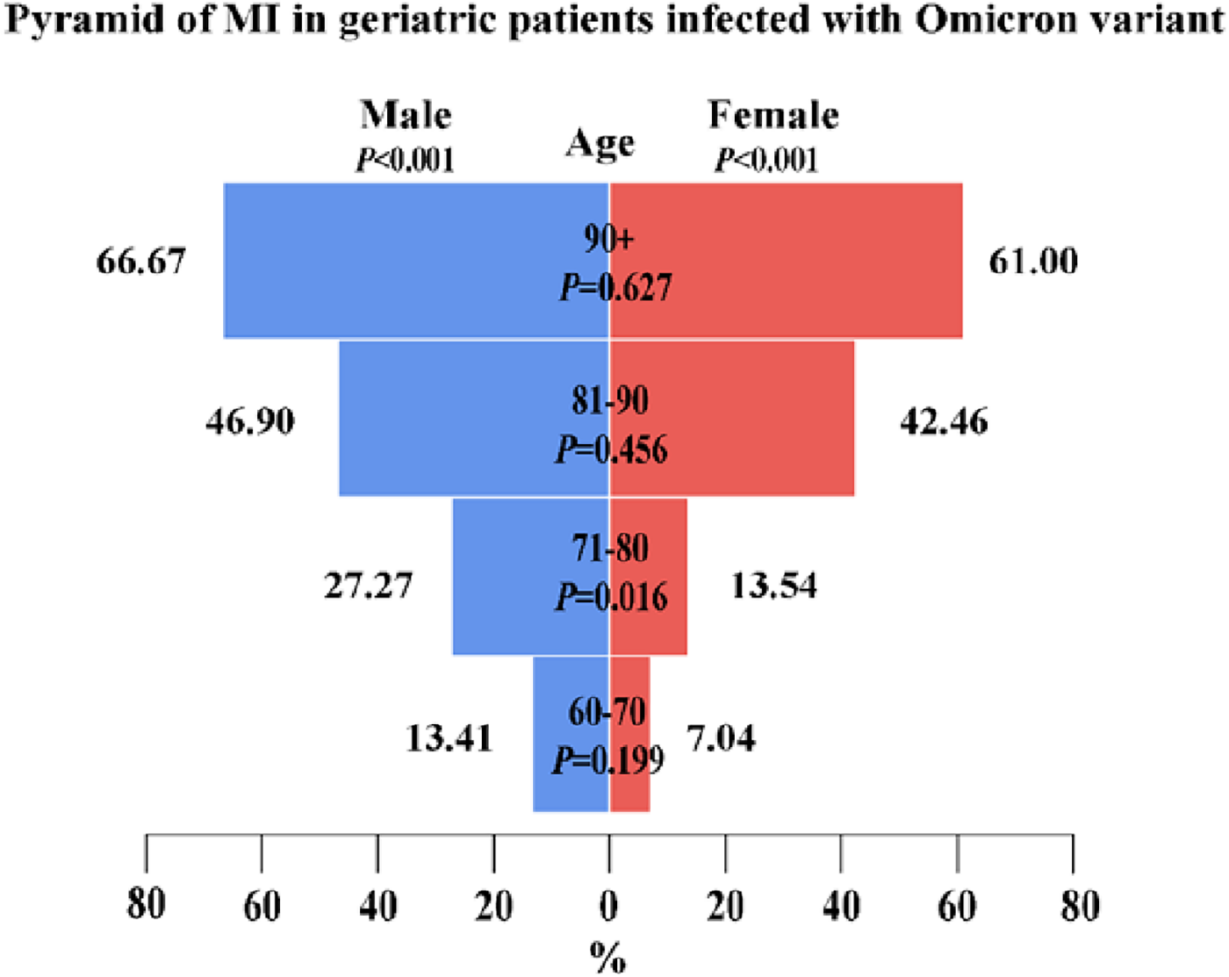
A pyramid of 263 elderly patients with myocardial injury among 772 elderly patients infected with the Omicron variant. The figure shows the age distribution of patients with myocardial injury among male and female patients and the gender distribution of patients with myocardial injury among those aged 60–70, 71–80, 81–90, and >90 years. A score of *P* < 0.05 was considered statistically significant.

Overall, patients with myocardial injury were older than those without myocardial injury [median (range), 87 (61–104) vs. 78 (60–101), respectively, *P* < 0.001]. Patients with myocardial injury were stratified by gender and age. The probability of myocardial injury increased with age in both male and female patients (*P* < 0.001). There was a significant difference in the gender distribution of patients with myocardial injury between 71 and 80 years (*P* = 0.016); however, there was no significant difference in the gender distribution of patients with myocardial injury among those aged 60–70, 81–90, and >90 years (Figure 2). Patients with myocardial injury were more likely to have shortness of breath and chest tightness than those without myocardial injury. Moreover, pre-existing diseases, including hypertension, coronary heart disease, cerebrovascular disease, arrhythmia, chronic kidney disease, and heart failure, were present more often among those with myocardial injury than those without myocardial injury (all *P*’s < 0.01) (Table 1).

### 3.2 Laboratory findings of Omicron variant–infected elderly patients with and without myocardial injury

Among all 772 patients, the median levels of D-dimer, interleukin-6, CRP, and SAA were all elevated, while the median levels of TP were decreased compared with normal values/ranges. The median levels of other pathological indicators, including WBC, NEUT, LYM, MON, and D-dimer, were within the normal range (Table 2). Patients with myocardial injury had higher median values of WBC (10^9^/L), NEUT (10^9^/L), NEUT%, hemoglobin, D-dimer, interleukin-6, procalcitonin, CRP, SAA, myoglobin, CK-MB, NT-proBNP, LDH, ALT, BUN, and sCr compared with those without myocardial injury, but neo-coronavirus Ct-ORF1ab values, Ct-N values, LYM (10^9^/L), LYM%, TP, and eGFR were lower.

**Table 2:** Clinical characteristics of Omicron variant–infected patients with and without myocardial injury.

Laboratory findings, median (IQR)/mean (±SD)			Myocardial injury		*P*-value
	*N*	Total (*n* = 772)	Present (*n* = 263)	Absent (*n* = 509)	
Ct-ORF1ab	766	21.13 (18.44–25.69)	20.22 (17.80–23.63)	21.61 (18.69–26.65)	<0.001
Ct-N	766	21.54 (18.71–25.98)	20.65 (18.30–24.15)	22.18 (19.06–26.81)	0.001
WBC, 10^9^/L	771	5.07 (4.00–6.66)	5.41 (4.18–7.08)	4.95 (3.93–6.24)	<0.001
NEUT, 10^9^/L	771	3.11 (2.19–4.50)	3.63 (2.44–5.68)	2.94 (2.12–4.14)	<0.001
NEUT, %	771	64.10 ± 13.99	68.22 ± 14.51	61.97 ± 13.24	<0.001
LYM, 10^9^/L	771	1.17 (0.85–1.59)	1.05 (0.73–1.45)	1.24 (0.92–1.68)	<0.001
LYM, %	771	25.42 ± 12.44	21.81 ± 12.51	27.28 ± 12.00	<0.001
MON, 10^9^/L	771	0.44 (0.32–0.58)	0.45 (0.34–0.61)	0.42 (0.32–0.57)	0.079
MON, %	771	8.70 (6.80–11.30)	8.50 (6.08–11.30)	8.80 (7.00–11.30)	0.126
Hemoglobin, g/L	771	124.00 (110.00–135.00)	119.00 (104.00–132.00)	126.00 (114.00–136.00)	<0.001
D-dimer, mg/L	719	0.65 (0.39–1.29)	1.05 (0.63–2.04)	0.50 (0.33–0.93)	<0.001
Interleukin-6, pg/ml	703	37.16 (19.44–127.60)	44.60 (25.90–149.18)	33.21 (16.42–119.40)	<0.001
Procalcitonin, ng/ml	733	0.024 (0.020–0.091)	0.075 (0.023–0.229)	0.020 (0.020–0.044)	<0.001
CRP, mg/L	768	10.71 (4.06–33.75)	21.45 (7.11–63.99)	8.06 (3.35–18.73)	<0.001
SAA, mg/L	672	39.05 (11.72–159.05)	74.81 (23.88–305.76)	26.18 (8.93–88.29)	<0.001
Myoglobin, ng/L	771	62.60 (44.71–99.13)	96.24 (66.70–229.48)	53.32 (39.94–70.98)	<0.001
CK-MB, ng/L	765	1.97 (1.63–3.47)	3.01 (1.83–4.86)	1.78 (1.56–2.86)	<0.001
NT-proBNP, pg/ml	665	269.60 (114.45–901.05)	1,022.00 (413.58–2,504.00)	165.30 (84.92–357.55)	<0.001
TP, g/L	754	60.95 ± 5.86	59.49 ± 5.95	61.66 ± 5.69	<0.001
Potassium, mmol/L	759	3.69 (3.38–4.01)	3.74 (3.39–4.12)	3.69 (3.38–3.96)	0.101
Sodium, mmol/L	759	143.00 (140.00–145.00)	142.00 (139.00–145.00)	143.00 (140.00–145.00)	0.056
Chlorate, mmol/L	759	105.00 (102.00–107.00)	105.00 (102.00–107.75)	105.00 (102.00–107.00)	0.478
LDH, U/L	623	201.38 (176.63–233.06)	218.58 (187.39–259.77)	195.23 (171.76–222.55)	<0.001
AST, U/L	756	16.40 (11.67–24.33)	16.42 (10.79–24.37)	16.38 (12.03–24.37)	0.417
ALT, U/L	756	24.91 (19.70–34.58)	27.81 (21.20–40.29)	23.74 (19.24–31.42)	<0.001
eGFR	757	98.00 (73.00–121.00)	80.00 (47.50–107.00)	104.50 (86.00–128.75)	<0.001
BUN, mmol/L	753	6.10 (4.80–8.25)	8.12 (5.80–11.98)	5.57 (4.47–7.14)	<0.001
sCr, μmol/L	757	62.00 (50.65–80.35)	75.20 (55.80–106.45)	58.55 (48.73–70.95)	<0.001

*N*, number of patients with available data.

Values are presented as mean ± SD for continuous variables with a normal distribution or median (IQR) for continuous variables without a normal distribution. Continuous variables were compared using *t*-tests or Mann–Whitney *U* tests, as appropriate. A score of *P* < 0.05 was considered statistically significant.

The cutoffs for laboratory examinations are as follows: WBC: 3.5–9.5 × 10^9^/L; NEUT: 1.8–6.3 × 10^9^/L; NEUT%: 40%–75%; LYM: 1.1–3.2 × 10^9^/L; LYM%: 20%–50%; Mono: 0.1–0.6 × 10^9^/L; Mono%: 3%–10%; hemoglobin: 120–150 g/L; D-dimer: ≤0.5 mg/L; interleukin-6: ≤6.6 pg/ml; procalcitonin: <0.5 ng/ml; CRP: 0–6 mg/L; SAA: 0–10 mg/L; myoglobin: male: 26.56–72.48 ng/L, female: 24.24–57.57 ng/L; CK-MB: male: ≤4.88 ng/L, female: ≤3.63 ng/L; NT-proBNP: <900 pg/mL; TP: 65–85 g/L; potassium: 3.5–5.3 mmol/L; sodium: 137–147 mmol/L; chlorate: 99–110 mmol/L; LDH: 120–250 U/L; ALT: male: 9–50 U/L, female: 7–40 U/L; AST: male: 15–40 U/L, female: 13–35 U/L; eGFR: 90–120; BUN: male: 3.6–9.5 mmol/L, female: 3.1–8.8 mmol/L; sCr: male: 57–111 μmol/L, female: 41–81 μmol/L.

### 3.3 Clinical treatment methods and outcomes of Omicron variant–infected elderly patients with and without myocardial injury

During hospitalization, 704 patients of those included in our study (91.19%) were treated with Paxlovid; the proportions of patients treated with heparin for anticoagulation, immunoglobulin, glucocorticoids, transnasal high-flow oxygen therapy, non-invasive ventilation, and invasive mechanical ventilation were 75.91% (*n* = 586), 5.05% (*n* = 39), 16.32% (*n* = 126), 4.53% (*n* = 35), 11.53% (*n* = 89), and 4.40% (*n* = 34), respectively. The proportions of mild, moderate, and severe cases were 43.52% (*n* = 336), 43.13% (*n* = 333), and 13.34% (*n* = 103), respectively. The median duration of hospitalization was 11 days (IQR, 8–15 days); during their hospital stay, 89 patients (23.30%) were transferred to the intensive care unit for treatment (Table 3).

**Table 3:** Treatment methods and clinical outcomes of patients with and without myocardial injury who were hospitalized with the Omicron variant.

Characteristic	Patients, No. (%)			
		Myocardial injury		
	Total (*n* = 772)	Present (*n* = 263)	Absent (*n* = 509)	*P-*value
Therapy				
Antivirus (Plaxlovid)	704 (91.19%)	238 (90.49%)	466 (91.55%)	0.623
Heparin	586 (75.91%)	213 (80.99%)	373 (73.28%)	0.018
Immunoglobulins	39 (5.05%)	14 (5.32%)	25 (4.91%)	0.805
Glucocorticoids	126 (16.32%)	67 (25.48%)	59 (11.59%)	<0.001
Transnasal high-flow oxygen therapy	35 (4.53%)	19 (7.22%)	16 (3.14%)	0.01
Non-invasive ventilation	89 (11.53%)	48 (18.25%)	41 (8.06%)	<0.001
Invasive mechanical ventilation	34 (4.40%)	20 (7.60%)	14 (2.75%)	0.002
Clinical outcomes				
Mild	336 (43.52%)	95 (36.12%)	241 (47.35%)	0.003
Moderate	333 (43.13%)	115 (43.73%)	218 (42.83%)	0.812
Severe	103 (13.34%)	53 (20.15%)	50 (9.82%)	<0.001
Hospitalization, median (IQR), days	11 (8–15)	13 (10–17)	10 (7–14)	<0.001
Duration of infection, median (IQR), days	13 (3–36)	15 (3–36)	12 (3–34)	<0.001
Intensive care	89 (23.30%)	53 (20.15%)	36 (7.07%)	<0.001

A higher proportion of patients with myocardial injury were given heparin therapy, glucocorticoid therapy, transnasal high-flow oxygen therapy, non-invasive ventilation, and invasive mechanical ventilation compared with those without myocardial injury (Table 2). Individuals with myocardial injury experienced both a prolonged duration of Omicron infection and an extended hospitalization compared with those without myocardial injury.

**Figure 3:**
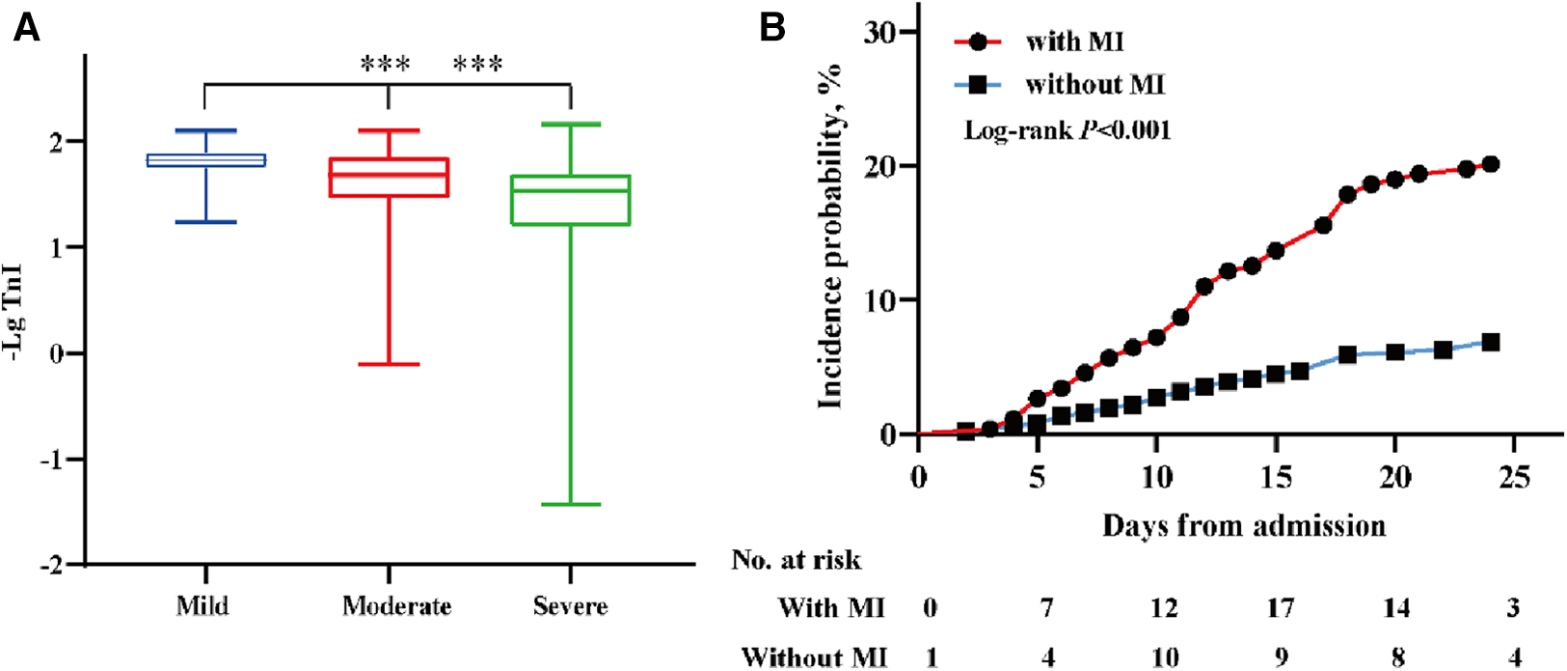
The distribution of TnI (-Lg) in different clinical classifications and the incidence probability of the endpoint event. (**A**) The distribution of TnI (-Lg) in subgroups (mild, moderate, severe) of patients according to the clinical classification; ***P < 0.001. (**B**) The incidence probability of patients transferred to the intensive care unit among patients with and without myocardial injury. A score of *P* < 0.05 was considered statistically significant.

Patients infected with the Omicron variant were divided into three groups (mild, moderate, and severe) according to the severity of their disease; those with severe disease had higher serum TnI levels than patients with mild or moderate disease (Figure 3A). A higher proportion of those with myocardial injury progressed to severe disease than those without myocardial injury (Table 3). Furthermore, patients with myocardial injury were more likely to be transferred to the intensive care unit for treatment than those without myocardial injury (*P* < 0.001) (Figure 3B).

### 3.4 Potential risk factors for myocardial injury in elderly patients infected with the Omicron variant

**Figure 4:**
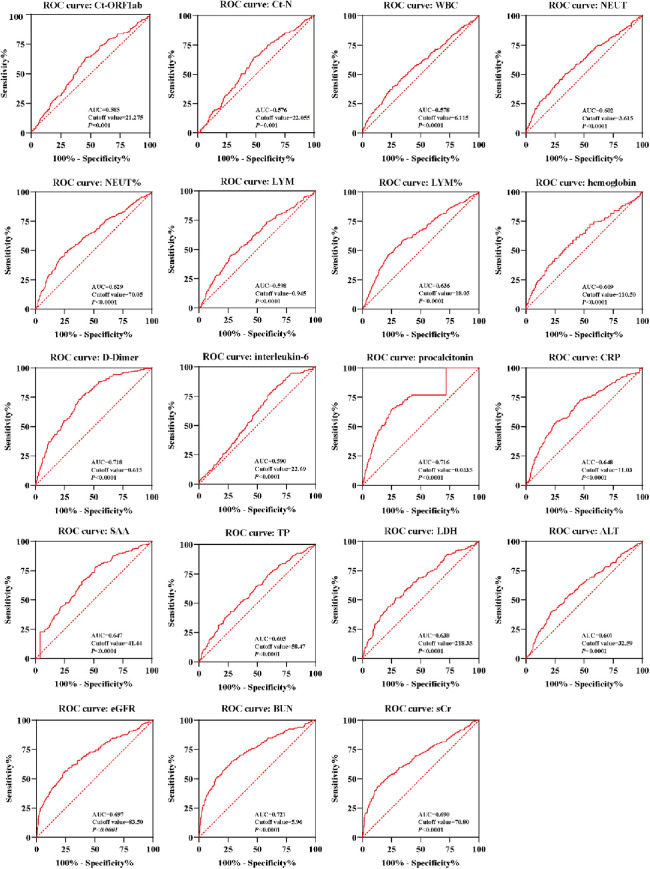
An ROC curve of laboratory indicators of Omicron variant–infected elderly patients with myocardial injury. ROC curves of the Ct-ORF1ab, Ct-N, WBC, NEUT, NEUT%, LYM, LYM%, hemoglobin, D-dimer, interleukin-6, procalcitonin, CRP, SAA, TP, LDH, ALT, eGFR, BUN, and sCr were calculated for Omicron variant–infected elderly patients with myocardial injury (*n* = 263) and without (*n* = 509) myocardial injury. AUC, area under the curve. A score of *P* < 0.05 was considered statistically significant.

An ROC curve analysis was performed to assess the predictive value of routine pathological indicators for myocardial injury, excluding myocardial indicators such as Ct-ORF1ab, Ct-N, WBC, NEUT, NEUT%, LYM, LYM%, hemoglobin, D-dimer, interleukin-6, procalcitonin, CRP, SAA, TP, LDH, ALT, eGFR, BUN, and sCr. The results, depicted in Figure 4, indicated that these indicators showed varying degrees of accuracy in predicting myocardial injury. The sensitivity and specificity of these indicators in predicting myocardial injury were both statistically significant (*P* < 0.05). The respective cutoff values for these indicators were determined to be within ranges, such as 21.275 for Ct-ORF1ab, 22.055 for Ct-N, 6.115 × 10^9^/L for WBC, 3.615 × 10^9^/L for NEUT, 70.05% for NEUT%, 0.945 × 10^9^/L for LYM, 18.05% for LYM%, 110.5 g/L for hemoglobin, 0.615 mg/L for D-dimer, 22.69 pg/ml for interleukin-6, 0.0435 ng/ml for procalcitonin, 19.15 mg/L for CRP, 21.33 mg/L for SAA, 58.47 g/L for TP, 218.065 U/L for LDH, 32.59 U/L for ALT, 83.5 for eGFR, 6.865 mmol/L for BUN, and 81.3 μmol/L for sCr.

Based on the optimal cutoffs determined by the ROC curve, the values of laboratory biomarkers were converted to categorical variables. As shown in Table 4, indicators with statistically significant differences included age, hypertension, coronary artery disease, cerebrovascular disease, arrhythmia, chronic kidney disease, and heart failure, and categorical variables for indicators were identified in the logistic regression analyses. In Table 4, the univariate logistic regression analyses revealed significant associations. The odds ratios (ORs) and 95% confidence intervals (CIs) were determined for various factors. All of these associations were statistically significant (*P* < 0.05). In the multivariable logistic regression analyses, several factors were identified as potential risk factors for myocardial injury. These included advanced age, pre-existing coronary heart disease, interleukin-6 levels >22.69 pg/ml, procalcitonin levels >0.0435 ng/ml, D-dimer levels >0.615 mg/L, and sCr levels >81.30 μmol/L. The corresponding ORs and 95% CIs were 1.063 (1.036–1.091), 2.171 (1.350–3.491), 2.101 (1.202–3.673), 3.199 (1.967–5.202), 2.108 (1.273–3.489), and 3.077 (1.841–5.141), respectively.

**Table 4:** Results of univariate and multivariate analyses of myocardial injury in 772 patients infected with the Omicron variant using logistic regression analyses.

Characteristic	Univariate			Multivariate		
	OR	95% CI	*P-*value	OR	95% CI	*P-*value
Age	1.091	1.072–1.111	<0.001	1.063	1.036–1.091	<0.001
Hypertension	1.802	1.313–2.472	<0.001	—	—	—
Coronary artery disease	2.746	1.994–3.780	<0.001	2.171	1.350–3.491	0.001
Arrhythmology	2.09	1.355–3.224	0.001	—	—	—
Chronic kidney disease	3.45	1.927–18.90	0.002	—	—	—
Cerebrovascular disease	1.635	1.183–2.260	0.003	—	—	—
Heart failure	6.036	1.959–6.078	<0.001	—	—	—
Ct-ORF1AB < 21.275	2.095	1.541–2.849	<0.001	—	—	—
Ct-N < 22.055	1.936	1.424–2.633	<0.001	—	—	—
WBC > 6.115 × 10^9^/L	1.85	1.347–2.541	<0.001	—	—	—
NEUT > 3.615 × 10^9^/L	2.007	1.481–2.720	<0.001	—	—	—
NEUT% > 70.05%	2.729	1.994–3.736	<0.001	—	—	—
LYM < 0.945 × 10^9^/L	2.157	1.578–2.949	<0.001	—	—	—
LYM% < 18.05%	3.042	2.207–4.193	<0.001	—	—	—
Hemoglobin < 110.5 g/L	2.444	1.753–3.407	<0.001	—	—	—
SAA > 21.33 mg/L	3.349	3.388–6.853	<0.001	—	—	—
CRP > 19.15 mg/L	3.465	1.638–3.456	<0.001	—	—	—
Interleukin-6 > 22.69 pg/ml	2.379	3.858–7.474	<0.001	2.101	1.202–3.673	0.009
Procalcitonin > 0.0435 ng/ml	5.37	2.526–4.755	<0.001	3.199	1.967–5.202	<0.001
TP < 58.47 g/L	2.024	2.335–4.803	<0.001	—	—	—
LDH > 218.065 U/L	2.679	1.472–2.783	<0.001	—	—	—
ALT > 32.59 U/L	2.400	1.726–3.337	<0.001	—	—	—
D-dimer > 0.615 mg/L	4.819	1.893–3.791	<0.001	2.108	1.273–3.489	0.004
eGFR < 83.5	4.124	2.985–5.699	<0.001	—	—	—
BUN > 6.865 mmol/L	4.821	3.487–6.667	<0.001	—	—	—
sCr > 81.3 μmol/L	5.105	3.566–7.308	<0.001	3.077	1.841–5.141	<0.001

A score of *P* < 0.05 was considered statistically significant.

In the univariate analysis, all items listed in the table were potential risk factors. In the multivariate analysis, advanced age, pre-existing coronary heart disease, interleukin-6 >22.69 pg/mL, procalcitonin >0.0435 ng/mL, D-dimer >0.615 mg/L, and sCr >81.30 μmol/L were potential risk factors; these laboratory biomarkers were analyzed as categorical variables.

## 4 Discussion

Elderly patients infected with the SARS-CoV-2 Omicron variant have gained attention in clinical care as they are at increased risk of death. Myocardial injury is a common complication associated with serious illness and death in those infected with SARS-CoV-2, especially in elderly patients with multiple comorbid chronic diseases (10–13). However, there has been little research on the epidemiology of myocardial injury in elderly patients infected with the Omicron variant. Reports on the early clinical identification and monitoring of myocardial injury among these patients are rare. In this study, we retrospectively analyzed clinical and laboratory data related to Omicron variant–infected elderly patients at the Fourth People's Hospital of Tongji University in Shanghai between 12 April and 17 June 2022. Our results showed that there were significant differences in clinical disease progression and related pathological indicators between those with and those without myocardial injury. Advanced age, coronary artery disease, and elevated levels of interleukin-6, procalcitonin, D-dimer, and sCr were considered potential risk factors for myocardial injury among elderly patients infected with the Omicron variant. This study will help clinicians to identify those with myocardial injury at hospital admission and to effectively manage and treat them.

The rate of incidence of myocardial injury in the epidemic caused by the wild-type SARS-CoV-2 has been reported to range between 7.2% and 36% (5, 11, 14–16), and myocardial injury has been shown to be common in patients with COVID-19. Our study showed that the proportion of myocardial injury in Omicron variant–infected elderly patients could be as high as 34.07%. Although the virulence and pathogenicity of the Omicron variant appear to be lower than that of the Alpha or Delta variants that preceded it, the elevated occurrence of myocardial injury among the aging population infected with the Omicron variant cannot be ignored. Evidence from several studies suggests that myocardial injury is an independent risk factor for adverse outcomes in patients (17), and the occurrence of myocardial injury is positively correlated with both exacerbation and death (12, 18–20). In our study, we found that a greater proportion of elderly patients with myocardial injury than those without had infections that became severe, requiring either non-invasive or invasive ventilatory respiratory support, and even transfer to intensive care. In addition, individuals with myocardial injury experienced an extended length of hospitalization compared with those without.

The occurrence of myocardial injury is usually found in elderly patients. Meanwhile, the proportion of myocardial injury is higher in patients with pre-existing conditions such as hypertension, coronary heart disease, cerebrovascular disease, arrhythmia, heart failure, and chronic kidney disease. A previous study reported that 63.5% of those with myocardial injury had hypertension, 32.7% had coronary artery disease, and 9.6% had chronic kidney disease (11). Similarly, other reported case series have demonstrated that those with SARS-CoV-2 complicated by myocardial injury have higher rates of pre-existing cardiovascular disease (CVD) (12, 20). Pre-existing CVD may also make patients more susceptible to myocardial infarction induced by SARS-CoV-2 (5, 20). Therefore, the early identification and prompt treatment of patients with pre-existing CVD are necessary.

SARS-CoV-2 can directly invade cardiomyocytes by binding to ACE2 receptors on the surface of cardiomyocytes (21–23). In real-world clinical studies, the replication of SARS-CoV-2 has been detected in the heart tissue (24–26), suggesting that SARS-CoV-2 can infect the myocardium. However, some pathological anatomical studies reported that only the infiltration of inflammatory cells, not the virus itself, was found in the cardiac tissue of patients who died from COVID-19 (27–29). This suggests that the occurrence of myocardial injury is likely caused by a systemic inflammatory response induced by SARS-CoV-2 infection. Whether SARS-CoV-2 directly damages the heart remains to be confirmed. Compared with previous studies on myocardial injury during the epidemic caused by the wild-type SARS-CoV-2, we increased the analysis of viral load indicators by including Ct-ORF1ab and Ct-N, which could be considered a first. The levels of these indicators were lower in those with myocardial injury compared with those without myocardial injury in this study. Furthermore, in our univariate regression analyses, we found that the levels of Ct-ORF1ab and Ct-N, along with those of inflammatory indicators, including interleukin-6, SAA, CRP, and procalcitonin, were significantly associated with the occurrence of myocardial injury. In the multifactorial regression analysis, neither Ct-ORF1ab nor Ct-N was found to be a statistically significant risk factor for myocardial injury. Although we cannot rule out that the virus directly damages cardiomyocytes, an excess of circulating inflammatory mediators produced by excessive systemic inflammatory responses is a more likely explanation for myocardial injury.

During the outbreak of wild-type SARS-CoV-2 in Wuhan, Shi et al. found that advanced age, pre-existing diseases (e.g., hypertension, coronary artery disease, chronic renal failure, and chronic obstructive pulmonary disease), and inflammatory markers (procalcitonin and CRP) were predictors of in-hospital myocardial injury among patients with severe COVID-19 (5). Li et al. also reported that advanced age (>70 years), CRP > 100 mg/L, LDH > 300 U/L, and lactate > 3 mmol/L were high-risk factors associated with myocardial injury in patients who were critically ill with COVID-19 (12). Consistent with these previous studies, we found that advanced age, pre-existing coronary artery disease, interleukin-6, and procalcitonin remain high-risk factors for the occurrence of myocardial injury in elderly patients infected with the Omicron variant. In addition, our study showed, for the first time, that D-dimer and sCr are also potential risk factors for myocardial injury in those infected with the Omicron variant. The pre-existing chronic conditions present among the elderly patients included in our study may have contributed to this result, as these patients are prone to microvascular dysfunction (30, 31). Some studies have found that the Omicron variant may directly attack vascular endothelial cells, leading to abnormal coagulation and microcirculatory disorders. Changes in blood flow tend to cause micro-myocardial injury, and subsequently, even lead to cardiac ischemia (32–34). Inflammation during infection with the Omicron variant also promotes endothelial dysfunction and accelerates procoagulant activity in the blood, which results in myocardial injury (35). There is evidence to suggest that COVID-19 can increase susceptibility for thromboembolic events such as deep vein thrombosis, pulmonary embolism, and even arterial *in situ* thrombosis (36). However, thromboembolic events were found to be infrequent in our study. Given the older age of our patient population, with a prevalence of cardiovascular chronic diseases, we implemented thromboprophylaxis measures. Heparin was widely utilized, achieving a coverage rate exceeding 75% among the patients recruited in our study. In addition, it is appropriate to consider adjusting the dosage of heparin or other anticoagulant drugs for patients identified as being at a high risk of thrombosis. Even a mild elevation of serum sCr is associated with an increased long-term risk of decompensated heart failure (37) or acute myocardial infarction (38, 39). A study in northern Italy found that patients with COVID-19 and heart disease had elevated serum sCr (40). Similarly, an epidemiological study of myocardial injury in patients intubated for COVID-19 found that TnI and sCr levels were highly positively correlated (41). Therefore, it is reasonable and necessary to pay attention to the occurrence of myocardial injury in elderly patients with abnormalities in D-dimer and sCr levels.

### 4.1 Limitations

This study has several limitations. First, it is a retrospective, single-center analysis; larger cohorts of older adults from multiple centers are needed to further explore the characteristics. Second, our analysis focused on myocardial injury within 24 h of admission. The dynamics of TnI levels and the timing of myocardial injury onset and its duration were not explored. Third, this study focused on the early identification of myocardial injury among older adults infected with the Omicron variant; some measures associated with cardiovascular diseases, such as lipid levels in the blood, were not included in the analysis because these values were not measured or recorded in many patients.

## 5 Conclusion

To conclude, there has been significant progress in the clinical treatment of patients infected with SARS-CoV-2; however, the occurrence of myocardial injury in elderly patients infected with the Omicron variant is an important factor determining clinical outcomes. Advanced age, coronary artery disease, interleukin-6 > 22.69 pg/ml, procalcitonin > 0.0435 ng/ml, D-dimer > 0.615 mg/L, and sCr > 81.30 μmol/L are potential risk factors of myocardial injury. The results of this study will enable healthcare professionals to identify those with myocardial injury at an early stage and to manage and treat such patients in an appropriate and timely manner to reduce disease severity and improve outcomes.

## Data Availability

The raw data supporting the conclusions of this article will be made available by the authors without undue reservation.
